# LncRNA VINAS regulates atherosclerosis by modulating NF-**κ**B and MAPK signaling

**DOI:** 10.1172/jci.insight.140627

**Published:** 2020-11-05

**Authors:** Viorel Simion, Haoyang Zhou, Jacob B. Pierce, Dafeng Yang, Stefan Haemmig, Yevgenia Tesmenitsky, Galina Sukhova, Peter H. Stone, Peter Libby, Mark W. Feinberg

**Affiliations:** 1 Cardiovascular Division, Department of Medicine, Brigham and Women’s Hospital, Harvard Medical School, Boston, Massachusetts, USA.; 2 Department of Cardiology, The Third Xiangya Hospital of Central South University, Changsha, Hunan, China.; 3 Feinberg School of Medicine, Northwestern University, Chicago, Illinois, USA.

**Keywords:** Vascular Biology, Atherosclerosis, Noncoding RNAs, endothelial cells

## Abstract

Long noncoding RNAs (lncRNAs) play important roles in regulating diverse cellular processes in the vessel wall, including atherosclerosis. RNA-Seq profiling of intimal lesions revealed a lncRNA, *VINAS* (Vascular INflammation and Atherosclerosis lncRNA Sequence), that is enriched in the aortic intima and regulates vascular inflammation. Aortic intimal expression of *VINAS* fell with atherosclerotic progression and rose with regression. *VINAS* knockdown reduced atherosclerotic lesion formation by 55% in LDL receptor–deficient (LDLR^–/–^) mice, independent of effects on circulating lipids, by decreasing inflammation in the vessel wall. Loss- and gain-of-function studies in vitro demonstrated that *VINAS* serves as a critical regulator of inflammation by modulating NF-κB and MAPK signaling pathways. *VINAS* knockdown decreased the expression of key inflammatory markers, such as MCP-1, TNF-α, IL-1β, and COX-2, in endothelial cells (ECs), vascular smooth muscle cells, and bone marrow–derived macrophages. Moreover, *VINAS* silencing decreased expression of leukocyte adhesion molecules VCAM-1, E-selectin, and ICAM-1 and reduced monocyte adhesion to ECs. DEP domain containing 4 (DEPDC4), an evolutionary conserved human ortholog of *VINAS* with approximately 74% homology, showed similar regulation in human and pig atherosclerotic specimens. DEPDC4 knockdown replicated antiinflammatory effects of *VINAS* in human ECs. These findings reveal a potentially novel lncRNA that regulates vascular inflammation, with broad implications for vascular diseases.

## Abstract 2

Neutralization of lncRNA VINAS or its human ortholog DEPDC4 limits the inflammatory response and atherosclerotic progression by regulating the NF-κB and MAPK signaling pathways.

## Introduction

Accumulating studies highlight that inflammatory processes and traditional cardiac risk factors may cooperatively contribute to vascular disease leading to the development of cardiovascular events (1, 2). More than 150 years ago Virchow hypothesized involvement of inflammation in atherosclerosis (3). However, the CANTOS trial only recently confirmed in humans the inflammatory hypothesis of atherosclerosis, by showing that neutralization of the proinflammatory cytokine IL-1β reduced recurrent cardiovascular events independent of changes in serum lipid levels (4, 5).

Inflammation impairs endothelial functions. For example, in response to both biochemical (e.g., IL-1β, modified LDL) and biomechanical (e.g., disturbed blood flow) stimuli, endothelial activation occurs early in atherogenesis (6). Expression of adhesion molecules (e.g., VCAM-1, E-selectin, ICAM-1) and secretion of chemokines (e.g., MCP-1) facilitate the recruitment of leukocyte subsets into the vessel wall (7). Impaired endothelial barrier function accompanies vascular inflammation and atherosclerosis (1, 8). Similar to ECs, smooth muscle cells (SMCs) can also express a variety of adhesion molecules in response to cytokine stimulation to which monocytes and lymphocytes can adhere and migrate into the vessel wall (5, 9, 10). However, major mechanistic gaps remain in our understanding of regulatory pathways involved in homeostasis of the vessel wall in response to pathophysiological stimuli, contributing to the dearth of targeted therapeutics in a range of vascular disease states.

Recently, long noncoding RNAs (lncRNAs) have emerged as powerful regulators of nearly all biological processes by exerting epigenetic, transcriptional, or translational control of target genes owing to their polyvalent binding properties to RNA, DNA, and protein as well as acting as molecular sponges for other transcripts and miRNAs (11, 12). However, the role of lncRNAs in vascular inflammation and cardiovascular diseases is just now emerging (13). Identification of lncRNAs specifically expressed in the vascular intima of lesions during the progression of atherosclerosis may provide insight into their roles in atherogenesis and potentially uncover new insight into vascular inflammation in advanced lesions (14).

This study identifies lncRNA *VINAS* (Vascular INflammation and Atherosclerosis lncRNA Sequence) as a key regulator of vascular inflammation and atherosclerotic lesion formation. We further find that its human ortholog, DEP domain containing 4 (DEPDC4), was similarly expressed in atherosclerotic lesions and phenocopied effects on human endothelial cell (EC) inflammation. Collectively, these findings provide new insight into lncRNA-mediated control of inflammation in the vessel wall.

## Results

### Identification and characterization of VINAS lncRNA.

**Figure 1:**
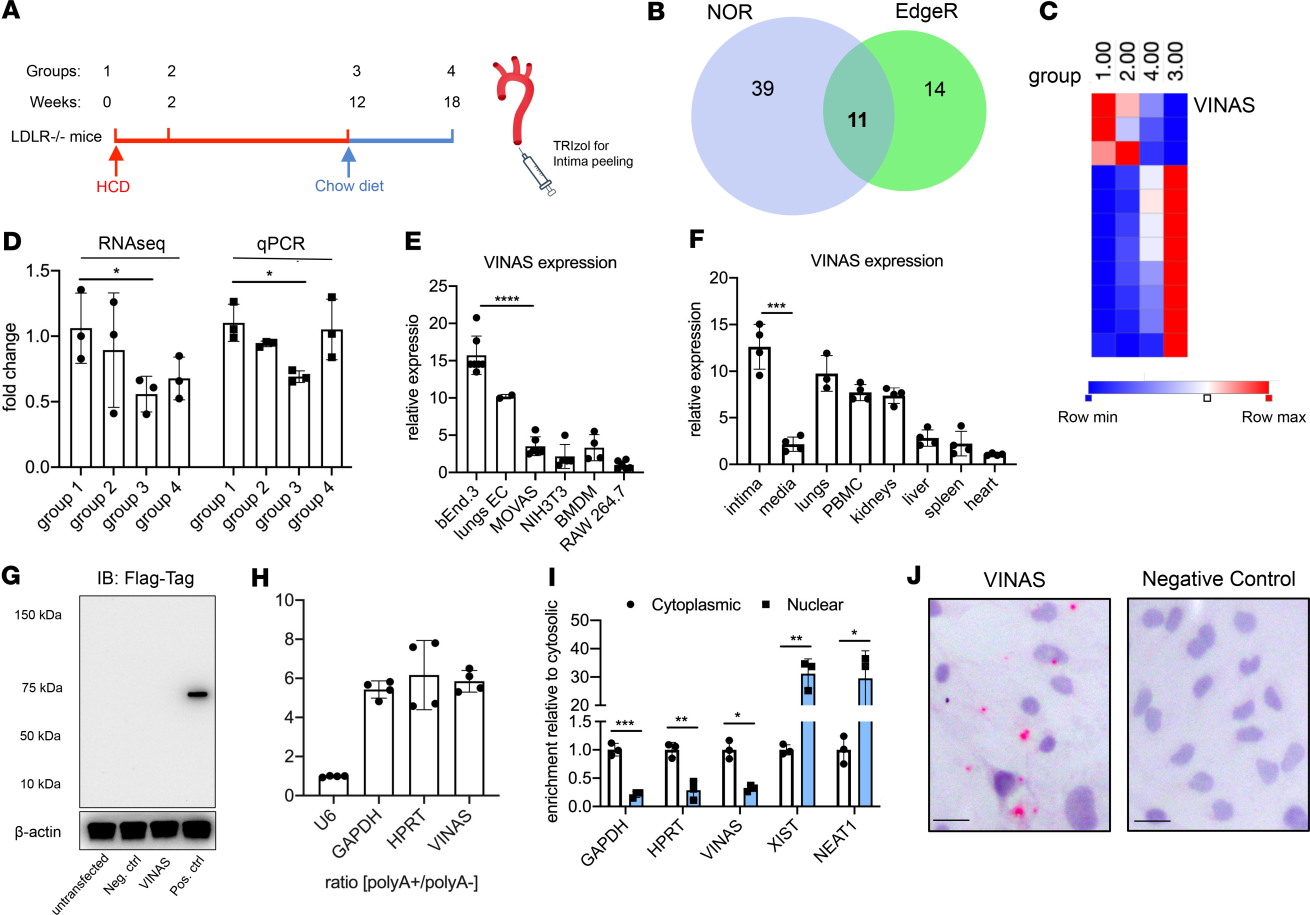
Identification of the lncRNA *VINAS* in lesional intima. (**A**) RNA derived from aortic intima of LDLR^–/–^ mice (*n* = 3; each sample represents RNA pooled from 2 mice) that were placed on a high-cholesterol diet (HCD) for 0 (group 1), 2 (group 2), 12 (group 3), and 18 weeks after 6 weeks of resumption of a normal chow diet (group 4). (**B**) Venn diagram displays significantly dysregulated lncRNAs in genome-wide RNA-Seq profiling using EdgeR and no-overlapping reads (NOR) showing intersecting hits (*n* = 11), uniquely identified in EdgeR (*n* = 14) or NOR (*n* = 39), (log_2_ fold change [1.5]; FDR < 0.05). (**C**) Heatmap for 11 lncRNAs that were dynamically regulated with progression and regression of atherosclerosis (*n* = 3). (**D**) RNA-Seq results for *VINAS* across groups 1–4 obtained by RNA-Seq analysis and verified by RT-qPCR (*n* = 3). (**E**) RT-qPCR expression analysis for *VINAS* in different cell types (*n* = 3). (**F**) *VINAS* expression in body organs and PBMCs of 24-week-old C57BL/6 mice (*n* = 4). (**G**) To test the coding potential, *VINAS* sequence was cloned upstream of 3xFlag-Tag cassette, transfected in HEK293T cells, and immunoblotted for Flag antibody. Positive control was provided with the kit (representative of 3 experiments). (**H**) RNA from mouse extracellular cells (ECs) was isolated for polyA^+^ and polyA^–^ enriched RNA and analyzed by real-time quantitative PCR (RT-qPCR) (*n* = 3). (**I**) RT-qPCR analysis for RNA derived from mouse ECs separated into cytoplasmic and nuclear fractions and normalized to the cytoplasmic fraction (*n* = 3). (**J**) RNA in situ hybridization for negative control and *VINAS* probes on paraformaldehyde-fixed mouse ECs. Scale bar: 5 μm. Data represent the mean ± SD. Statistical differences were calculated using unpaired 2-tailed Student’s *t* test except for multiple comparisons (**E** and **F**) in which 1-way ANOVA with Bonferroni’s correction was used. *P < 0.05, **P < 0.01, ***P < 0.001.

LDL receptor–deficient (B6.129S7-*Ldlr^tm1Her^*/J; LDLR^–/–^) male mice were placed on a high-cholesterol diet (HCD) for 0, 2, and 12 weeks (progression phases, groups 1–3) and subsequently placed on a chow diet for another 6 weeks in the fourth group (regression phase, Figure 1A). RNA was isolated from the aortic intima and RNA-Seq profiling revealed 11 differentially expressed lncRNAs (log_2_ fold change 1.5; FDR < 0.05) using EdgeR and no overlapping reads (NOR) algorithms (Figure 1B). Eight lncRNAs rose with atherosclerosis progression (group 3) and fell during regression (group 4), whereas only 3 lncRNAs decreased with atherosclerosis progression (Figure 1C). The lncRNA 1500026H17Rik showed the strongest decrease in group 3 (by 59%), while regaining initial levels with atherosclerosis regression as quantified by real-time quantitative PCR (RT-qPCR) (Figure 1, C and D). Because of its high regulability and participation in both vascular inflammation and atherosclerosis, as we show here, we have named this lncRNA *VINAS* (Vascular INflammation and Atherosclerosis lncRNA Sequence).

Further experiments characterized arterial *VINAS* expression. *VINAS* expression was higher in ECs (isolated from lungs and b.End.3 cell line) compared with other cell types, such as vascular smooth muscle cells (VSMCs) (MOVAS cell line), NIH3T3 fibroblasts, bone marrow–derived macrophages (BMDMs), or the RAW 264.7 macrophage cell line (Figure 1E), and was broadly expressed in several other organs, with a strong enrichment in the aortic intima compared with the media in the vessel wall (Figure 1F). Our previous study verified the specificity of aortic intima isolation (15). Therefore, to test whether *VINAS* lncRNA encodes any protein or peptide, the *VINAS* sequence was cloned upstream of the p3xFLAG-CMV plasmid, transfected in HEK293 cells, and immunoblotted for FLAG Tag, yielding no detectable peptide or protein (Figure 1G). Additionally, *VINAS* was found to be polyadenylated (Figure 1H) and enriched in the cytosol, as observed by cellular fractionation and by RNA-ISH in mouse ECs (Figure 1, I and J).

### VINAS regulates inflammation in vascular cells.

**Figure 2:**
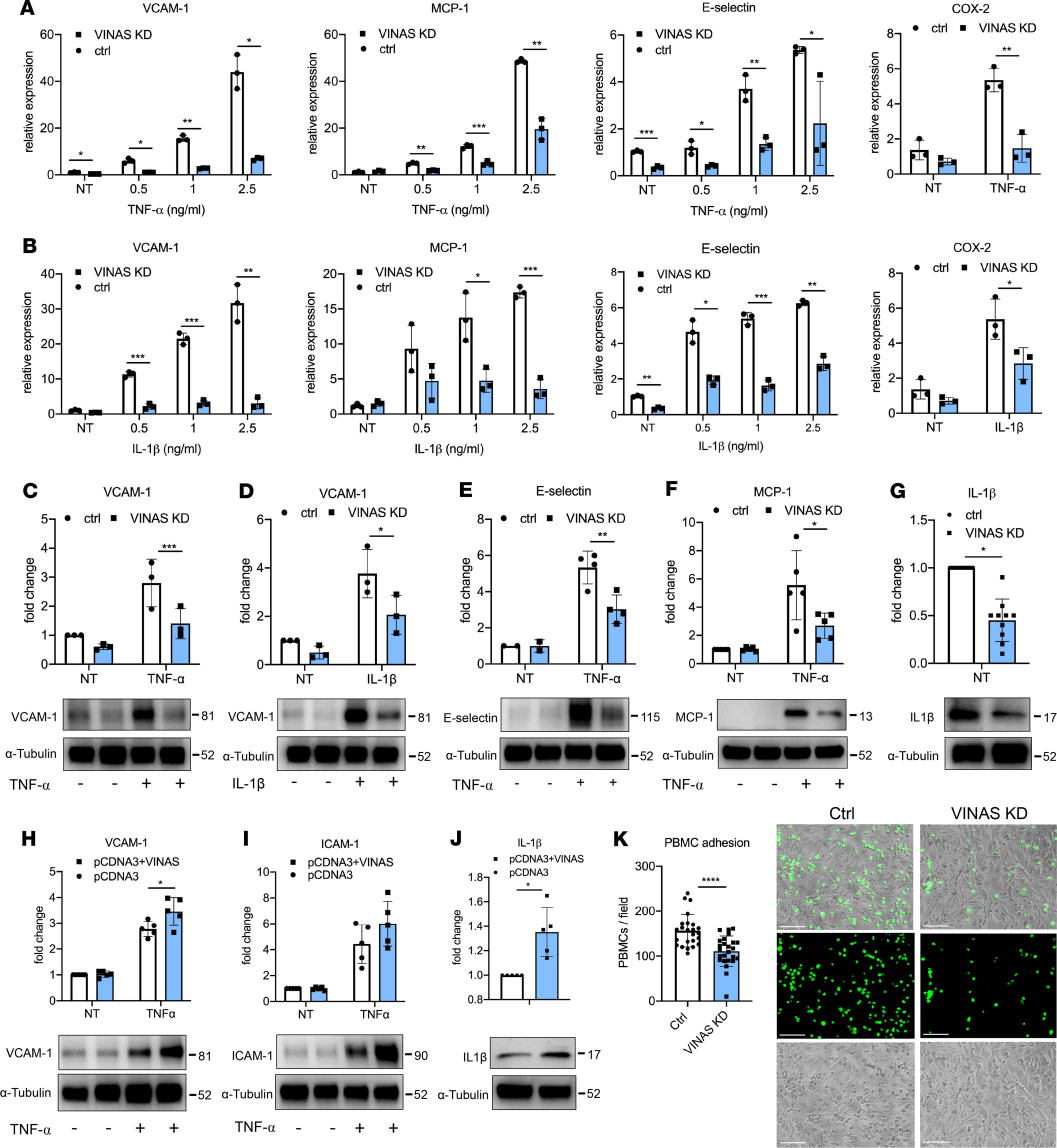
*VINAS* regulates inflammatory markers in endothelial cells. *VINAS* knockdown decreases the mRNA levels of VCAM-1, E-selectin, MCP-1, and COX2 in mouse ECs activated with TNF-α (**A**) and IL-1β (**B**); *n* = 3. *VINAS* silencing decreases the protein expression of VCAM-1 (**C** and **D**, *n* = 3), E-selectin (**E**, *n* = 4), MCP-1 (**F**, *n* = 5), and IL-1β (**G**, *n* = 10) in basal conditions or after activation with 20 ng/mL TNF- or IL-1β. *VINAS* overexpression increases the protein expression of VCAM-1 (**H**), ICAM-1 (**I**), and IL-1β (**J**) in mouse ECs not treated or activated with 20 ng/mL TNF-α (*n* = 5). (**K**) *VINAS* knockdown decreases the PBMCs’ adhesion to mouse ECs activated with TNF-α for 4 hours (5 ng/mL, representative of 3 experiments). Scale bar: 50 μm. Data represent the mean ± SD. Statistical differences were calculated using unpaired 2-tailed Student’s *t* test. *P < 0.05, **P < 0.01, ***P < 0.001, ****P < 0.0001.

**Figure 3:**
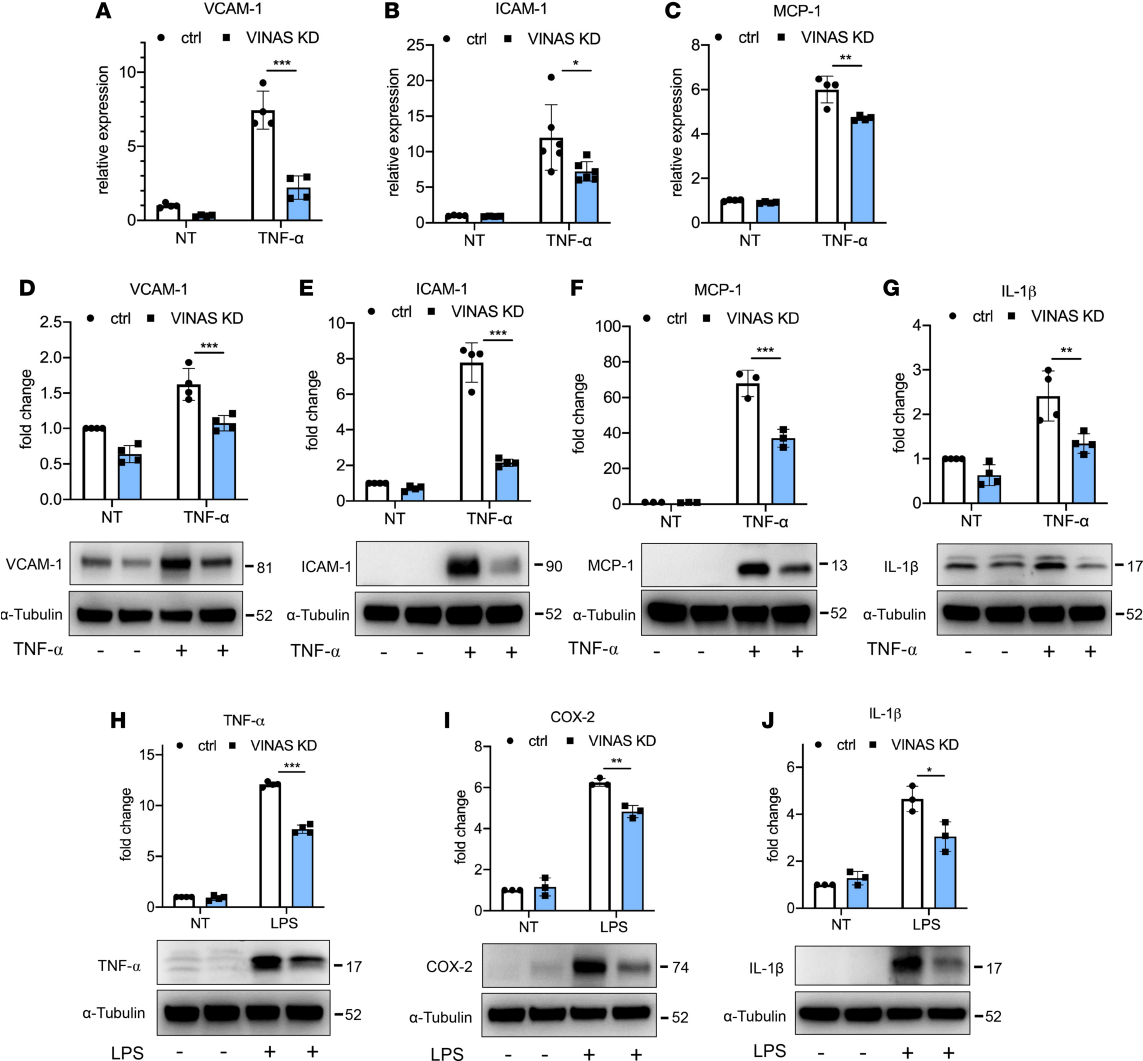
*VINAS* knockdown decreases inflammation in SMCs and BMDMs. *VINAS* knockdown decreases mRNA levels of VCAM-1 (**A**, *n* = 4), ICAM-1 (**B**, *n* = 6), and MCP-1 (**C**, *n* = 4) in MOVAS smooth muscle cells (SMCs) stimulated with 5 ng/mL TNF-α. *VINAS* knockdown decreases protein expression of VCAM-1 (**D**, *n* = 4), ICAM-1 (**E**, *n* = 4), MCP-1 (**F**, *n* = 3), and IL-1β (**G**, *n* = 4) in MOVAS smooth muscle cells stimulated with 20 ng/mL TNF-α. *VINAS* knockdown decreases the protein expression of TNF-α (**H**), COX-2 (**I**), and IL-1β (**J**) in bone marrow–derived macrophages (BMDMs) stimulated with 50 ng/mL LPS (*n* = 3). Data represent the mean ± SD. Statistical differences were calculated using unpaired 2-tailed Student’s *t* test. *P < 0.05, **P < 0.01, ***P < 0.001.

ECs participate pivotally in vascular inflammation and development of atherosclerosis. Because *VINAS* is enriched in ECs (Figure 1E), the potential phenotype of *VINAS* loss- and gain-of-function was assessed in mouse ECs. For the knockdown strategy, we designed 3 different locked nucleic acid (LNA) gapmeRs without any significant effect on apoptosis as observed by detection of Caspase 3 cleavage (Supplemental Figures 1B and 3; supplemental material available online with this article; https://doi.org/10.1172/jci.insight.140627DS1) GapmeR 2 showed the highest silencing efficiency in a dose-dependent manner (Supplemental Figure 1C), and it was used throughout the study. The gapmeR-mediated knockdown of *VINAS* dramatically decreased the mRNA expression of the adhesion molecules VCAM-1 by 50%–95% and E-selectin by approximately 40%–65% in ECs activated with 0.5, 1, and 2.5 ng/mL TNF-α or IL-1β (Figure 2, A and B). In addition, *VINAS* knockdown in activated ECs reduced the mRNA expression of the chemokine MCP-1 by approximately 50%–80% and the inflammatory molecule COX-2 by approximately 40%–55% (Figure 2, A and B). Moreover, *VINAS* silencing produced similar effects at the protein level, decreasing VCAM-1 by 45%–55% after activation with 20 ng/mL TNF-α or IL-1β (Figure 2, C and D), and MCP-1, COX-2, and IL-1β by approximately 50% (Figure 2, E–G). Transfection with 2 different *VINAS* gapmeRs (gapmeR 1 and 3) produced comparable decreases in VCAM-1 and COX-2 in ECs activated with 20 ng/mL TNF-α (Supplemental Figure 1E). In contrast, *VINAS* overexpression using a pCDNA3 plasmid (Supplemental Figure 1D) had the opposite effect in mouse ECs, increasing the protein expression of VCAM-1 (20%), ICAM-1 (26%), and IL-1β (35%) (Figure 2, H–J). Because *VINAS* knockdown in ECs decreased the expression of VCAM-1 and E-selectin, 2 cell adhesion molecules known to mediate leukocyte adhesion to ECs, we assessed adhesion of PBMCs to EC monolayers in response to 10 ng/mL TNF-α stimuli. *VINAS* knockdown reduced PBMCs adherence to EC monolayers by 29% (*P* < 0.0001), verifying the functional importance of *VINAS* lncRNA in leukocyte-EC cellular interactions (Figure 2K). Further experiments assessed the antiinflammatory actions of *VINAS* in 2 other cell types that are enriched in atheroma: VSMCs and BMDMs. We observed similar effects of *VINAS* knockdown in the MOVAS SMC line, with reduced expression of VCAM-1 (70%), ICAM-1 (40%), and MCP-1 (22%) at the mRNA level and decreased protein expression of VCAM-1 (34%), ICAM-1 (72%), MCP-1 (22%), TNF-α (37%), and IL-1β (44%) after stimulation with 5 ng/mL TNF-α (Figure 3). Consistently, *VINAS* silencing also decreased COX-2 (19%), IL-1β (38%), and MCP-1 (37%) in primary BMDMs stimulated with 50 ng/mL LPS (Figure 3). Collectively, these findings indicate that *VINAS* broadly regulates inflammatory mediators in relevant cell types in the vessel wall. The stronger antiinflammatory phenotype observed in ECs compared with VSMCs and BMDMs correlated with the increased *VINAS* expression in ECs (Figure 1E).

### VINAS regulates NF-κB and MAPK signaling pathways in ECs.

**Figure 4:**
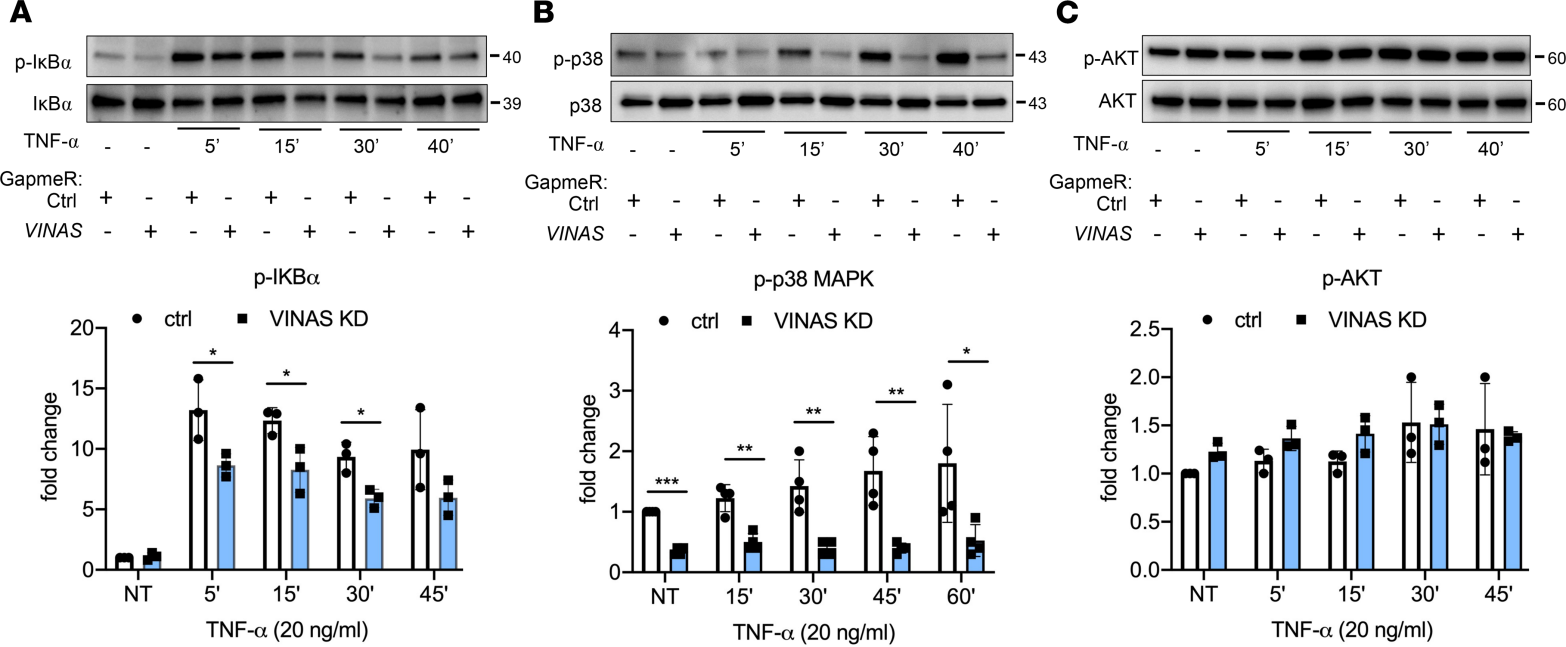
*VINAS* knockdown regulates NF-κB and p38 MAPK signaling pathways. Mouse ECs were transfected with *VINAS* gapmeRs and activated with TNF-α (20 ng/mL) for 5, 15, 30, 45, and 60 minutes. Phosphorylation of IκBα (**A**, *n* = 3), p38 MAPK (**B**, *n* = 4), and AKT (**C**, *n* = 3) was assessed by Western blot. Data represent the mean ± SD. Statistical differences were calculated using 1-way ANOVA with Bonferroni’s correction. *P < 0.05, **P < 0.01, ***P < 0.001.

To identify potential signaling pathways subject to *VINAS* regulation, ECs transfected with *VINAS* gapmeRs were activated with 20 ng/mL TNF-α for 5–60 minutes and assessed for expression of key proinflammatory signaling pathways. Immunoblotting showed that *VINAS* knockdown significantly decreased the phosphorylation of IκBα in ECs activated with TNF-α (20 ng/mL) by 35%, 33%, and 37% after 5, 15, and 30 minutes, respectively (Figure 4A). In addition, silencing of *VINAS* in ECs reduced the phosphorylation of p38 MAPK by 55%–75% in the presence of TNF-α (20 ng/mL) for 15, 30, and 45 minutes (Figure 4B). Similar conditions were tested for AKT signaling pathway and showed no specific effect of *VINAS* silencing on AKT phosphorylation (Figure 4C). Taken together, these findings indicate that *VINAS* knockdown regulates predominantly the NF-κB and MAPK signaling pathways.

### In vivo knockdown of VINAS markedly reduced atherosclerotic lesion formation by decreasing vascular inflammation.

**Figure 5:**
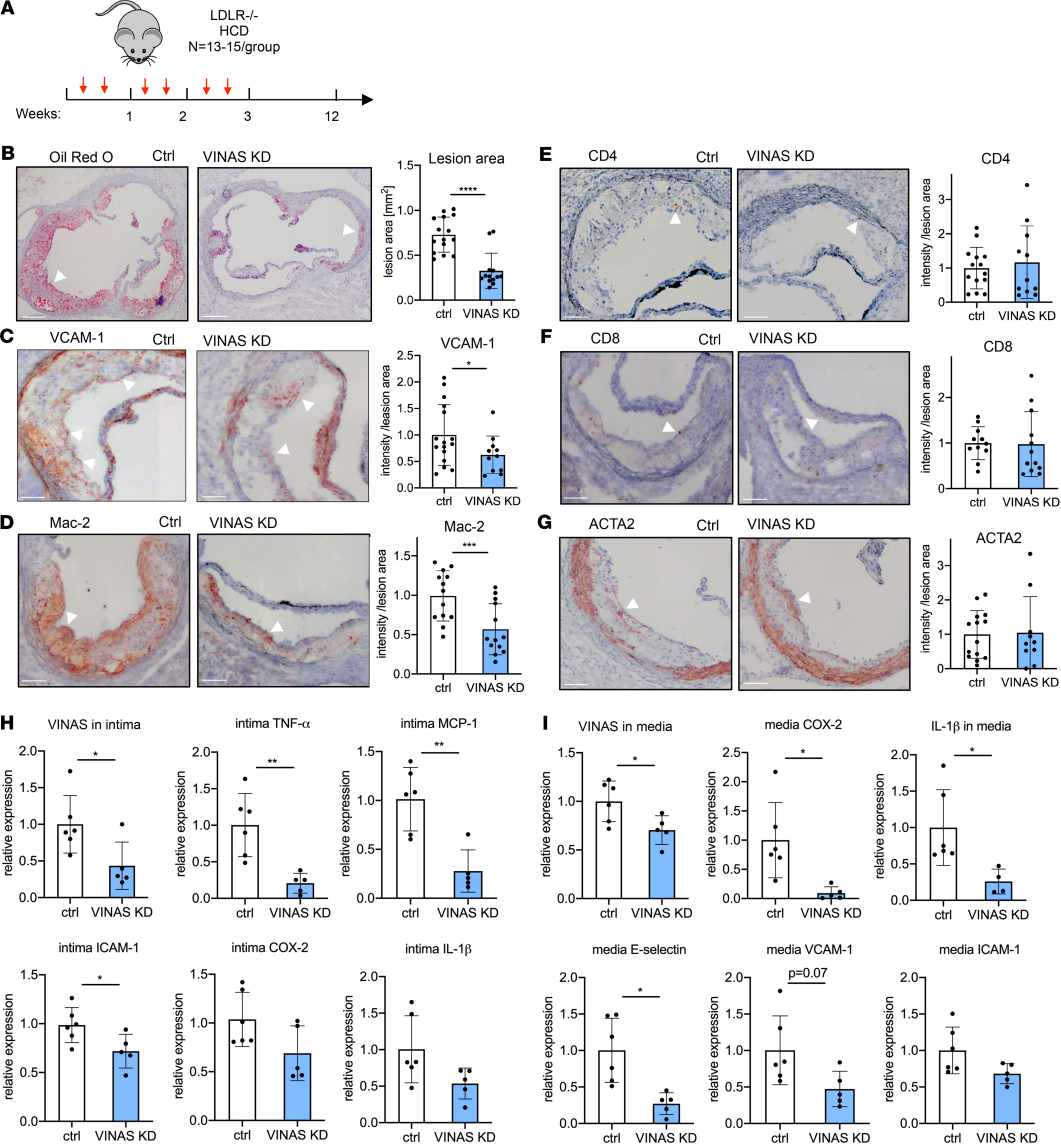
In vivo knockdown of *VINAS* inhibits atherosclerotic lesion formation by decreasing vascular inflammation. (**A**) LDLR^–*/*–^ mice were i.v. injected with vehicle control gapmeR (*n* = 15) or *VINAS* gapmeR (*n* = 13) twice per week (10 mg/kg/mouse/injection) and placed on an HCD for 12 weeks. Representative images and quantification for Oil Red O (scale bar: 400 μm) (**B**), VCAM-1 (**C**), Mac-2 (**D**), CD4^+^ (**E**), CD8^+^ (**F**), and ACTA2 (**G**) staining (arrowhead) of the aortic sinus of LDLR^–/–^ HCD mice treated with control (*n* = 15) or *MAARS* (*n* = 13) gapmeRs for 12 weeks. Scale bar: 100 μm. *VINAS* silencing efficiency and expression of inflammatory markers was assessed by RT-qPCR in the intima (**H**) and media (**I**) fractions of the aortic arch from control gapmeR (*n* = 6) and *VINAS* gapmeR groups (*n* = 5). Data represent the mean ± SD. Statistical differences were calculated using unpaired 2-tailed Student’s *t* test. *P < 0.05, **P < 0.01, ***P < 0.001, ****P < 0.0001.

To explore whether systemically delivered *VINAS* gapmeRs modulate atherosclerosis, LDLR^–/–^ mice received i.v. injections of vehicle control or *VINAS* gapmeR (10 mg/kg/2 times weekly) over 12 weeks on an HCD (Figure 5A). After 12 weeks on an HCD, gapmeR-mediated silencing of *VINAS* reduced its expression in the aortic intima by 57% (Figure 5H) and in the media by 30% (Figure 5I).

Analysis of atherosclerotic lesion formation by Oil Red O (ORO) staining revealed a 55% decrease in lesion area in the aortic sinus after antagonism of *VINAS* (Figure 5B). Although *VINAS* knockdown was associated with a modest reduction in total cholesterol (22%), LDL (25%), HDL (6%), and triglycerides (7%) (Supplemental Figure 2A), the lesion areas as quantified by ORO staining remained 48% smaller in *VINAS* knockdown mice when examined in mice, with similar total cholesterol in both groups (Supplemental Figure 2B). Although approximately 8% of the atherosclerotic plaque reduction may be accounted for effects on cholesterol metabolism, it cannot account entirely for the marked reduction in atherosclerosis lesions following *VINAS* knockdown.

IHC staining revealed that VCAM-1 and the macrophage marker Mac-2 decreased by 38% and 43%, respectively, in the aortic sinus, indicating reduced vascular inflammation and macrophage accumulation in the vascular wall (Figure 5, C and D). No significant differences were observed for CD4^+^ or CD8^+^ T cells or VSMCs after normalization to lesion area (Figure 5, E–G). In vivo knockdown of *VINAS* in the aortic intima reduced the expression of inflammatory markers TNF-α, MCP-1, ICAM-1, COX-2, and IL-1β (Figure 5H). Moreover, *VINAS* knockdown in the aortic media decreased inflammatory effectors, such as COX-2, IL-1, E-selectin, VCAM-1, and ICAM-1 (Figure 5I). Although *VINAS* silencing also reduced circulating PBMCs (62%), it did not significantly alter mRNAs that encode the inflammatory mediators TNF-α, IL-1β, COX-2, and MCP-1 in these cells (Supplemental Figure 1F). Nor did *VINAS* knockdown alter the antiinflammatory Ly6C^lo^ or the proinflammatory Ly6C^interm^ or Ly6C^hi^ fractions in the PBMCs as determined by flow cytometry (Supplemental Figure 1G). Overall, *VINAS* neutralization in LDLR^–/–^ mice fed an HCD for 12 weeks muted atherosclerotic lesion formation in tandem with decreased inflammation.

### DEPDC4 is a VINAS ortholog conserved in humans.

**Figure 6:**
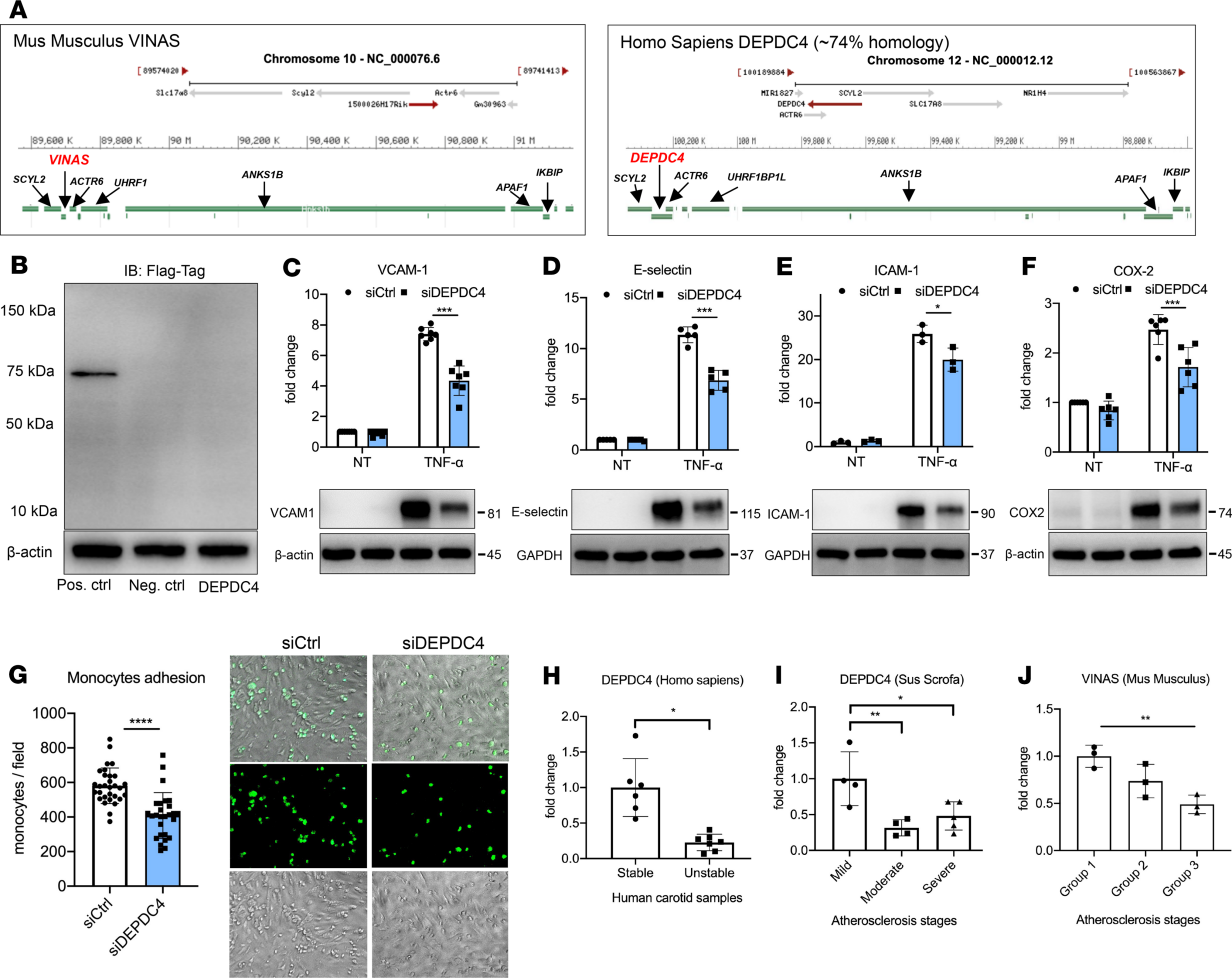
DEPDC4 is a human ortholog of VINAS. (**A**) Illustration of the genomic locations of *VINAS* and DEPDC4 in the mouse and human chromosomes 10 and 12, respectively. (**B**) DEPDC4 does not encode for a protein or peptide. To test the coding potential, DEPDC4 sequence was cloned upstream of the 3xFlag-Tag cassette, transfected in HEK293T cells, and immunoblotted for Flag antibody; positive control was provided with the kit (*n* = 3 experiments). DEPDC4 silencing decreases the protein expression of VCAM-1 (**C**, *n* = 7), E-selectin (**D**, *n* = 5), and ICAM-1 (**E**, *n* = 3) COX-2 (**F**, *n* = 6) in HUVECs activated with 20 ng/mL TNF-α. (**G**) DEPDC4 knockdown decreases THP-1 monocyte adhesion to HUVEC monolayers activated with TNF-α for 4 hours (5 ng/mL, representative images and quantification of adhered monocytes). (**H**) RT-qPCR of DEPDC4 in human carotid arteries with stable (*n* = 6) or unstable (*n* = 7) atherosclerotic plaques. Scale bar: 50 μm. (**I**) Expression of DEPDC4 from RNA-Seq analyses of lesions with increasing severity of coronary atherosclerosis in Yorkshire pigs fed an HCD for 60 weeks (*n* = 4/group). (**J**) RT-qPCR of *VINAS* expression in aortic intima of LDLR^–/–^ mice at 0, 2, and 12 weeks of an HCD (*n* = 3/group). Data represent the mean ± SD. Statistical differences were calculated using unpaired 2-tailed Student’s *t* test except for multiple comparisons (**I** and **J**) in which 1-way ANOVA with Bonferroni’s correction was used. *P < 0.05, **P < 0.01, ***P < 0.001, ****P < 0.0001.

Although *VINAS* lncRNA is only present in the mouse genome, we observed that the genomic locus is largely conserved, with the genes SCYL2, ACTR6, and ANKS1B in the immediate proximity and the gene DEPDC4 in the same position as *VINAS* (Figure 6A). BLAST (http://www.ncbi.nlm.nih.gov/blast/) findings showed that DEPDC4 has an approximately 74% homology with *VINAS* in a region of 157–323 bp, depending on isoform variations (Supplemental Figure 4A). DEPDC4 is widely conserved across species, except for the mouse (Supplemental Figure 4A). To verify the coding probability, the DEPDC4 sequence was cloned upstream of the p3xFLAG-CMV plasmid, transfected in HEK293 cells, and immunoblotted for the FLAG Tag. The resulting immunoblot showed no detectable peptide or protein (Figure 6B). As with *VINAS* loss of function in mouse cells, DEPDC4 knockdown (Supplemental Figure 4B) induced an antiinflammatory program in HUVECs stimulated with TNF-α, decreasing the expression of VCAM-1 (42%), E-selectin (40%), and COX-2 (30%) (Figure 6, C–E). We then assessed adhesion of THP-1 monocytes to a monolayer of HUVECs in response to TNF-α stimulation. DEPDC4 silencing significantly decreased monocyte adherence to the EC monolayer by 30%, verifying the functional importance of DEPDC4 lncRNA in leukocyte-EC interactions (Figure 6F).

**Figure 7:**
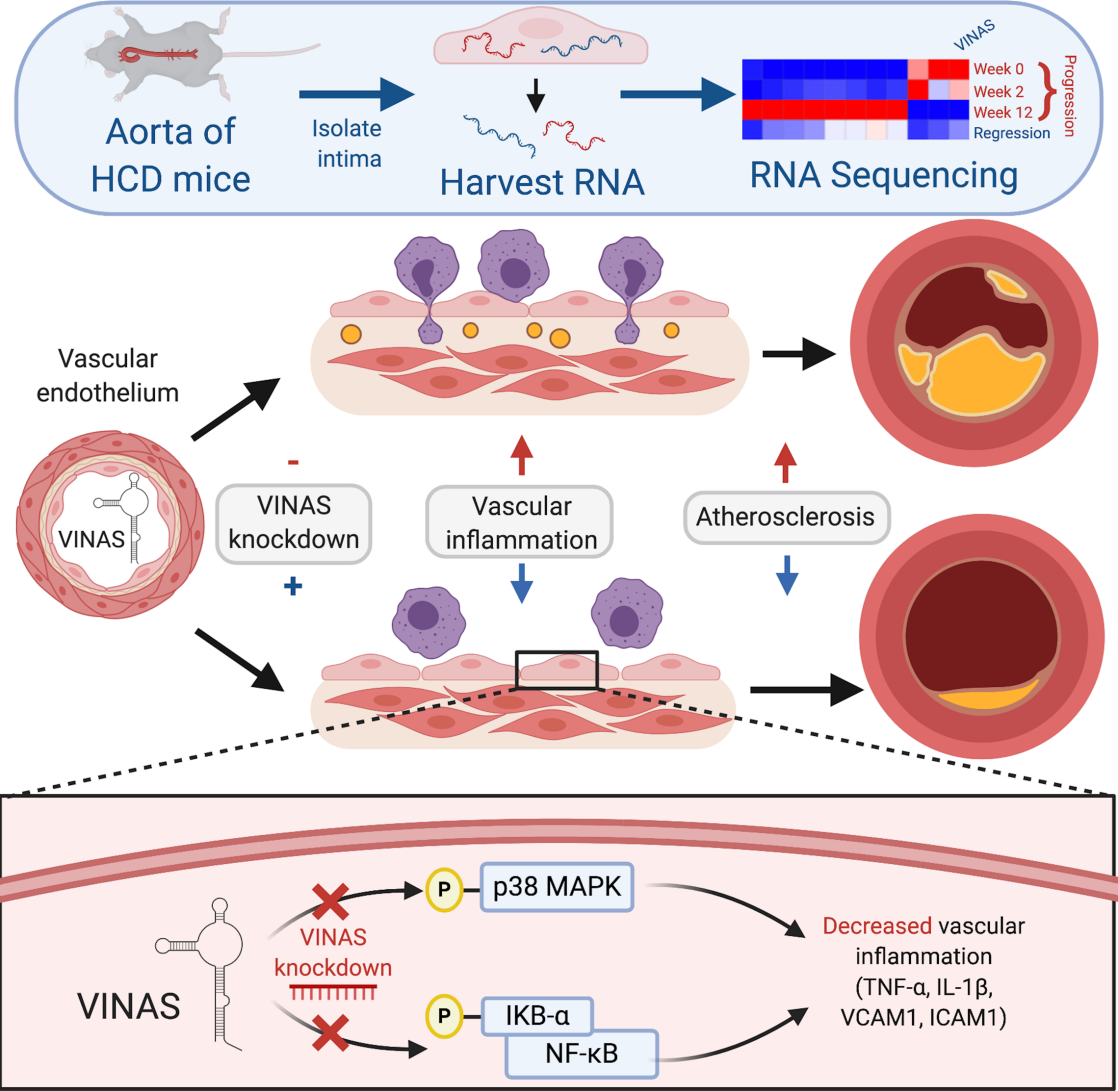
Summary of the role of lncRNA *VINAS* in vascular inflammation. RNA-Seq profiling of intimal lesions revealed *VINAS* lncRNA that is enriched in the aortic intima, decreased with atherosclerotic progression, and increased with regression. *VINAS* knockdown decreased the expression of key inflammatory markers, NF-κB and MAPK signaling pathways, cell adhesion molecules, and the monocytes adhesion to ECs. In vivo *VINAS* knockdown reduced atherosclerotic lesion formation in LDLR^–/–^ mice by decreasing vascular inflammation.

To assess the translational relevance of *VINAS* and DEPDC4 lncRNAs, RNA was isolated from human carotid atherosclerotic plaques with characteristics associated with stability or instability. DEPDC4 expression decreased by 77.4% in carotid arteries with plaques with unstable versus those with stable features (Figure 6G). To explore this expression pattern across species, we analyzed the RNA-Seq data from Yorkshire pigs that were placed for up to 60 weeks on an HCD and developed coronary atherosclerosis. Based on histopathological characterization, the coronary sections were separated into mild, intermediate, and severe groups for progression of atherosclerosis as previously described (15). Similar to *VINAS* regulation in LDLR^–/–^ mice fed an HCD (Figure 1C), DEPDC4 decreased approximately 60% with disease progression in swine pigs fed an HCD (Figure 6H). Concordantly, in ECs stimulated with TNF-α *VINAS* and DEPDC4 expression also decrease after 4–8 hours and 16–24 hours, respectively (Supplemental Figure 1, H and I). In summary (Figure 7), these results demonstrate dynamic regulation of the lncRNA *VINAS* with atherosclerosis progression, that *VINAS* influences arterial inflammation, and that loss of function of *VINAS*’s evolutionary conserved lncRNA ortholog DEPDC4 exerts similar antiinflammatory effects.

## Discussion

Arterial inflammation occurs very early in atherogenesis and is associated with impairment of many salutary functions of the healthy endothelium. Accumulating studies point to lncRNAs as regulators of endothelial homeostasis, smooth muscle cell contractility, and macrophage-mediated inflammation in the vessel wall (11, 13, 15–18). This study provides evidence for the first time to our knowledge that the mouse-specific lncRNA *VINAS* and its human ortholog, DEPDC4, play important roles in vascular inflammation and atherogenesis.

Our study expands upon a growing body of literature implicating lncRNAs as pivotal regulators in the development and progression of atherosclerosis. Our group recently identified SNHG12 as an evolutionary conserved lncRNA that plays an important role in atherogenesis (15). SNHG12 mediated the interaction between DNA damage repair proteins DNA-PK and its binding partners Ku70 and Ku80. Following *SNHG12* knockdown in LDLR^–/–^ mice, atherosclerotic lesion area increased by 240%, with corresponding increases in markers of DNA damage and EC senescence (15). The lncRNAs *LeXis* and *MeXis* were identified as key regulators of cholesterol metabolism (19, 20). Both of these lncRNAs are transcriptionally regulated by the liver X receptor, a nuclear sterol receptor responsible for transcriptional control of genes involved with cholesterol metabolism. *LeXis* interacted with the ribonuclear protein RALY to aid in transcription of cholesterol metabolism genes in the liver, and in vivo delivery of *LeXis* using an adenoviral vector reduced aortic atherosclerosis in mice (21). *MeXis* altered *ABCA1* expression via its binding partner DDX17, and genetic abrogation of *MeXis* increased serum cholesterol and atherosclerotic lesion area (20).

Similar to *VINAS*, several other lncRNAs regulate atherosclerosis by modulating inflammatory pathways. For example, the lncRNA *NEXN-AS1* lies antisense to and increases the expression of NEXN, a protein that negatively regulates TLR4 and NF-κB signaling (22). Genetic depletion of *NEXN-AS1* dramatically increased atherosclerosis in *ApoE*^–*/*–^ mice, with concurrent increases in markers of vascular inflammation such as VCAM-1, ICAM-1, TNF-α, and MCP-1. Similarly, knockdown of *lncRNA-FA2H-2* increased atherosclerotic plaque size and expression of inflammatory genes. Here, we show that *VINAS* plays an analogous role in inflammation and atherogenesis, albeit as a proinflammatory lncRNA in contrast to the antiinflammatory lncRNAs *NEXN-AS1* or *lncRNA-FA2H-2*. In vivo delivery of *VINAS*-specific LNA gapmeRs markedly decreased the expression of important inflammatory mediators and cell adhesion molecules in the intima as well as the media of the aortic arch. *VINAS* silencing exerted strong antiinflammatory effects across different cellular constituents of the vessel wall, demonstrated by decreased key inflammatory effectors such as MCP-1, TNF-α, IL-1β, COX-2, and the leukocyte adhesion molecules VCAM-1, E-selectin, or ICAM-1, in both ECs and VSMCs (Figure 2 and Figure 3). The stronger antiinflammatory phenotype observed in ECs and the intima is likely attributed to increased *VINAS* silencing efficiency (Figure 5, H and I; and Supplemental Figure 1, B and C) coupled with the relatively higher expression of VINAS in ECs and intima (Figure 1, E and F) compared with the aortic media. Also, the aortic media is composed of more heterogeneity of cell types (e.g., fibroblasts, VSMCs, and immune cells), and *VINAS* expression is variable across these different cell types (Figure 1E).

Leukocyte adhesion to activated ECs overexpressing adhesion molecules such as VCAM-1 and E-selectin is among the earliest processes involved in atherosclerotic lesion initiation (23, 24). This study shows that *VINAS* knockdown in TNF-α–activated ECs significantly reduced monocyte adhesion to EC monolayers (Figure 2K). In line with this finding, in vivo *VINAS* knockdown decreased the staining of macrophage marker Mac-2 in the aortic root, suggesting a diminished macrophage accumulation in the vessel wall attributed to lower expression of cell adhesion molecules (Figure 5D). Macrophage polarization to a proinflammatory phenotype contributes to the progression and destabilization of atherosclerotic plaques. For example, symptomatic patients suffering from acute transient ischemic attacks with unstable plaques had a higher concentration of M1 proinflammatory macrophages in lesions compared with asymptomatic patients with stable plaques (25, 26). Although the M1/M2 macrophage dichotomy oversimplifies macrophage heterogeneity, an M1 proinflammatory macrophage predominance in atherosclerotic plaques associates with a higher incidence of ischemic stroke and increased lesional inflammation (27). Moreover, plaques from patients with recently symptomatic carotid disease have a predominance of M1 macrophages and higher lipid content than femoral plaques, consistent with a more unstable plaque (28). Although *VINAS* knockdown in BMDMs in vitro decreased the expression of MCP-1, IL-1β, and COX-2 (Figure 3K), there were no differences in these effectors or of Ly6C^+^ proinflammatory PBMCs in vivo, suggesting that the antiinflammatory effects of *VINAS* knockdown in vivo were likely driven more by affecting leukocyte adhesion molecules in intimal ECs (Figure 2 and Figure 3). Although the dominant impact of lncRNA VINAS knockdown is regulating inflammation in the vessel wall, with a 48% reduction in atherosclerotic plaque when cholesterol values are normalized between the groups (Supplemental Figure 2B), we cannot exclude a minor contribution to cholesterol metabolism.

Identification of the potential signaling pathways that lncRNAs regulate is critical from a therapeutic point of view. In some cases, deciphering the signaling pathway and its upstream or downstream regulators can indicate the mechanisms used by a specific lncRNA (29). In this study, *VINAS* and DEPDC4 knockdown in cytokine-activated ECs reduced the phosphorylation of IκBα and p38 MAPK while having no significant effects on phosphorylation of the AKT signaling pathway (Figure 4). Both the NF-κB and p38 MAPK inflammatory pathways serve as critical nodal points of regulation in atherosclerosis, particularly in the vascular endothelium (30–32). Previously, Gareus et al. demonstrated that endothelium-specific genetic depletion of IKKγ or IκBα, key signaling molecules in the NF-κB pathway, was sufficient to significantly reduce atherosclerosis in *ApoE^–/–^* mice (33). Systemic delivery of miRNAs that inhibit NF-κB activation in the vascular endothelium also reduced inflammation and atherosclerosis lesions in *ApoE^–/–^* mice (34). Similarly, p38 MAPK inhibitors decreased levels of systemic and vascular inflammation in both mouse models of atherosclerosis (35, 36) as well as humans with coronary artery disease (37, 38). Furthermore, Seeger et al. demonstrated that systemic p38 MAPK inhibition for 4 weeks reduced atherosclerotic lesion size by more than 50% (36). Our study extends these findings by showing that lncRNA *VINAS* is an important regulator of NF-κB and p38 MAPK signaling pathways and thus exerts considerable control over the development of vascular inflammation and atherosclerosis. The observed antiinflammatory phenotype induced by *VINAS* knockdown may inform the potential upstream mechanisms by which this lncRNA affects these inflammatory pathways. *VINAS* lncRNA is enriched in the cytosol, and its knockdown potently reduced the phosphorylation of p38 MAPK, a signaling pathway with its main effectors localized in the cytosol (39–43). Although cytosolic lncRNAs have been reported to interact with miRNAs by a bp-binding mechanism (44, 45), this competing endogenous RNA (ceRNA) hypothesis remains controversial in the field. An in vivo quantitative study showed that modulation of a miRNA target abundance is unlikely to cause significant effects on gene expression and metabolism through a ceRNA effect (46). Future studies exploring the candidate factor(s) mediating this inhibition of dual signaling pathways may further elucidate potential therapeutic targets for atherosclerosis and other chronic inflammatory disease states.

Finally, whereas lncRNAs are not typically as conserved across species compared with other noncoding RNAs, such as miRNAs, emerging studies demonstrate conservation via orthologous transcripts (20). Finding an evolutionary conserved transcript DEPDC4, a human ortholog of *VINAS* in humans with approximately 74% homology, exhibited regulation in human EC cells congruent to the effects of *VINAS* on mouse cells supports the human relevance of the present mouse findings. Consistent with *VINAS* regulation in atherosclerotic mice, DEPDC4 levels declined in coronary arteries of pigs with progression of atherosclerosis and in human carotid plaques with unstable characteristics (Figure 6, G–I). *VINAS* expression also decreases in ECs after 4 and 8 hours’ incubation with TNF-α, while returning to basal levels after 16–24 hours (Supplemental Figure 1H). In all our experiments, the cytokines were added to the cells at 48 hours after gapmeRs’ transfection, when the *VINAS* silencing efficiency was already achieved by approximately 90%. Hence, the potential *VINAS* downregulation after cytokines’ addition would be negligible. Several mechanisms can be responsible for the observed *VINAS* regulation, including compensatory or feedback mechanisms in response to stress induced by inflammatory stimuli. For instance, LPS induces inflammation via the NF-κB pathway. However, LPS also induces the synthesis of antiinflammatory cytokines such as IL-10 and IL-4, which in turn blocks NF-κB activation in a negative feedback mechanism (47, 48), suggesting that the upregulation of antiinflammatory genes is not always coincident with inflammatory state. Indeed, lncRNAs can be regulated as a negative feedback mechanism during inflammation. For example, LPS increases the expression of lncRNA Mirt2. However, lncRNA Mirt2 serves as a negative feedback regulator of excessive inflammation and reduces inflammation across different cell types (49). Interestingly, the IL-10 antiinflammatory phenotype is regulated by the ubiquitously expressed transcription factor Sp1 (50), which also has multiple binding sites in the *VINAS* and DEPDC4 promoters (Supplemental Table 2). Although we have not identified the exact mechanism for the upstream regulation of *VINAS* lncRNA, we cannot rule out the existence of a compensatory mechanism in response to proinflammatory stimuli. Future studies will need to assess the specific upstream mechanism of *VINAS*/DEPDC4 regulation at the promoter and the transcript levels and whether this is a regulatory effect on RNA stability or a compensatory mechanism in the cell.

In conclusion, the discovery of *VINAS* reported here extends the understanding of participation of lncRNAs in inflammatory signaling in general and in the pathogenesis of atherosclerosis and potentially other vascular diseases as well. Modulation of lncRNAs *VINAS* and DEPDC4 may facilitate fine-tuning of the inflammatory response in a range of chronic vascular diseases and perhaps of other organ systems as well.

## Methods

### RNA-Seq analysis.

RNA-Seq analysis was performed after ribodepletion and standard library construction using Illumina HiSeq2500 V4 2x100 PE (Genewiz). All samples were processed using an RNA-Seq pipeline implemented in the bcbio-nextgen project (https://bcbio-nextgen.readthedocs.org/en/latest/). Raw reads were examined for quality issues using FastQC (http://www.bioinformatics.babraham.ac.uk/projects/fastqc/) to ensure library generation and sequencing were suitable for further analysis. Trimmed reads were aligned to UCSC build mm10 of the mouse genome, augmented with transcript information from Ensembl release 79 using STAR (51). Alignments were checked for evenness of coverage, rRNA content, genomic context of alignments (for example, alignments in known transcripts and introns), complexity, and other quality checks using a combination of FastQC and Qualimap. Counts of reads aligning to known genes were generated by featureCounts (52). Differential expression at the gene level was called with EdgeR. The total gene hit counts and counts per million values were calculated for each gene, and downstream differential expression analysis between specified groups was performed using EdgeR and an adapted EdgeR algorithm, which excludes overlapping reads, called NOR. Genes with adjusted FDR of less than 0.05 and log_2_ fold change 1.5 were called as differentially expressed genes for each comparison. Mean quality score of all samples was 35.67 within a range of 40,000,000–50,000,000 reads per sample. All samples had at least 70% or more mapped fragments over the total. RNA-Seq data are available through the NCBI’s Gene Expression Omnibus database (GSE138219).

### Polyadenylation.

RNA of 10^6^ ECs was isolated using TRIzol reagent (Invitrogen) and resuspended in RNase-free water. Polyadenylated and nonpolyadenylated RNA were enriched with the polyA Spin mRNA isolation kit (New England Biolabs, S1560S) based on the manufacturer’s protocol. Real-time PCR (RT-qPCR) was performed with same input volume, independent of concentration, and normalized to nonpolyadenylated RNA fraction.

### RNA-ISH.

Customized probe for *VINAS* was specifically developed to detect ENSMUST00000181598 (Advanced Cell Diagnostics). BMDMs were fixed in 4% paraformaldehyde, and the in situ hybridization protocol for cultured adherent cells was performed as described by the manufacturer (Basescope 2.5 HD Reagent Kit-Red; Advanced Cell Diagnostics).

### Protein coding potential.

Transcripts for *VINAS* (1500026H17Rik, NCBI Ref. Seq. NR_130956.1, Ensemble ID ENSMUST00000181598) were synthesized by Genewiz. For in vitro validation of peptide coding potential, *VINAS* transcript was cloned upstream of p3xFLAG-CMV-14 expression vector (MilliporeSigma, E7908) using EcoRI restriction site. HEK293T cells (ATCC, CRL-11268) were transfected with 500 ng plasmid using Lipofectamine 2000 (Invitrogen), and protein lysate was isolated 72 hours after transfection, followed by immunoblotting for FLAG Tag (Cell Signaling Technology, 8146).

### Molecular cloning for VINAS overexpression.

For overexpression studies, the *VINAS* transcript synthesized by Genewiz was cloned in a pCDNA.3 plasmid using the EcoRI restriction site. The integration was validated by DNA sequencing. For transfection studies in ECs, 0.25 μg plasmid/well (12-well plate) was used in combination with Lipofectamine 3000, according to the manufacturer’s instructions.

### Cell culture and transfection.

Mouse ECs (b.End.3, ATCC, CRL-2299), MOVAS mouse aortic SMCs (ATCC, CRL-279), and RAW 264.7 cells (ATCC, TIB-71) were cultured in DMEM with 10% FBS and 1% penicillin/streptomycin. HUVECs (Lonza, cc-2159) were cultured in EC growth medium EGM-2 (Lonza, cc-3162). Cells passaged less than 7 times were used for all experiments. Bone marrow was isolated from the femur and tibia of C57BL/6 mice and cultured in IMDM supplemented with 10 ng/mL mouse macrophage colony stimulation factor (416ML, R&D Systems, Bio-Techne), 10% FBS, and 1% penicillin/streptomycin. Medium was changed every 2 days, and cells were used in experiments after 7–10 days in culture. Transfection was performed using Lipofectamine 3000 (Invitrogen, 11668-019) according to the manufacturer’s protocol, and customized gapmeRs for *VINAS* (QIAGEN, 25 nmol except when mentioned differently) or negative control 1 (QIAGEN). Cells were allowed to grow for 36 hours before treatment with recombinant human TNF-α (210-TA/CF, R&D Systems, Bio-Techne), IL-1β (401-ML, R&D Systems, Bio-Techne), or LPS (O26:B6 *Escherichia coli*; MilliporeSigma L2654) for various times, according to the experiment: Western blot, 16 hours; RT-qPCR, 6 hours.

### Cell adhesion assay.

ECs grown in 24-well plates were transfected with gapmeRs. After 35 hours, 20 ng/mL TNF-α was added for 5 hours. PBMCs were isolated from C57BL/6 mice, washed, and suspended at 5 × 10^6^ cells/mL in medium with 5 μM Calcein AM (C3100MP; Invitrogen). Cells were kept in an incubator containing 5% CO_2_ at 37°C for 30 minutes. The labeling reaction was stopped by the addition of the cell growth medium, and cells were washed with growth medium twice and resuspended in growth medium at 5 × 10^5^ cells/mL. After 4 hours of TNF-α treatment, ECs were washed once with DMEM growth medium, and 500 μL Calcein AM–loaded PBMCs were added to each well. After 1 hour of incubation, nonadherent cells were removed carefully. Adherent cells were gently washed with prewarmed DMEM 4 times and were counted using a Nikon fluorescence microscope (Eclipse TE2000-U).

### RNA isolation and RT-qPCR.

Tissues were homogenized using TissueLyser II (QIAGEN) according to the manufacturer’s instructions. For RNA isolation, TRIzol reagent (Invitrogen) or RNeasy kit (QIAGEN) was used based on the manufacturer’s protocol. Isolation of intimal RNA and subsequent RT-qPCR from aorta was performed as previously documented (34, 53).

Briefly, aortas were carefully flushed with PBS, followed by intima peeling using TRIzol reagent (Invitrogen, 15596018). TRIzol was flushed for 10 seconds, followed by a 10-second pause, then for another 10 seconds flushed, collected in an Eppendorf tube (~300–400 μL total), and snap frozen in liquid nitrogen. The intima-specific isolation was assessed by qPCR showing enrichment of endothelial marker CD31 and macrophage marker Mac2 in the intima fraction compared with the media/adventitia fraction as previously described (15). Subsequent RT-qPCR was performed using High-Capacity cDNA Reverse Transcription kit (Applied Biosystems, 4368813). GoTaq qPCR Master Mix (Promega, A6001) was used for RT-qPCR experiments. Expression of mRNAs and lncRNA expression levels were normalized to GAPDH, HPRT, or β-actin (Agilent, AriaMx Real-Time PCR System). Changes in expression were calculated using the ΔΔCt method. Primer sequences are described in Supplemental Table 1.

### Cellular fractionation.

EC fractionation for cytoplasmic and nuclear fractions was performed using the Active Motif kit (catalog 40410) according to the manufacturer’s protocol. RNA was harvested as described previously and cleaned up using the RNeasy kit (QIAGEN). Equivalent RNA volumes of cytoplasmic and nuclear-associated RNA were converted to cDNA as described previously.

### Western blot.

Proteins were isolated using RIPA buffer (Boston BioProducts, BP-115) with protease inhibitor (Roche, 4693132001) and phosphatase inhibitors (New England Biolabs, P0758L). Protein concentrations were determined using Pierce BCA assay (Thermo Fisher Scientific). A total of 20 μg protein were loaded per lane on a 4%–20% Mini-PROTEAN TGX Gel (Bio-Rad, 456-1096). Separated proteins were transferred to PVDF membranes using the Transfer Turbo Blot system (Bio-Rad) and Trans-Blot Turbo RTA Transfer Kit (Bio-Rad, 170-4272). The membrane was blocked with 5% nonfat milk in TBST for 1hour at room temperature. After blocking, the membrane was incubated overnight at 4°C with antibodies against Flag Tag (Cell Signaling Technology, 2368, 1:1000), GAPDH (Cell Signaling Technology, 2118, 1:4000), VCAM-1 (Cell Signaling Technology, sc-13160, 1:1000), ICAM-1 (R&D Systems, Bio-Techne, BBA3), IκBα (Cell Signaling Technology, 4812, 1:1000), β-actin (Cell Signaling Technology, 4970, 1:3000), and phospho-IκBα (Cell Signaling Technology, 2859, 1:1000), IL-1β (Abcam ab9722, 1:1000), MCP-1 (Abcam ab25124, 1:1000), COX-2 (Cell Signaling Technology 12282p), p-P38MAPK (Cell Signaling Technology 4511L, 1:1000), and P38 MAPK (Cell Signaling Technology 9212L, 1:1000). Quantification of protein bands was performed using a luminescent image analyzer (Bio-Rad, Chemidoc).

### Immunohistology and characterization of atherosclerotic lesions.

To quantify atherosclerosis in LDLR^–/–^ mice that were placed on a HCD (Research Diets Inc., D12108C), aortic roots and aortic arch were embedded in OCT and frozen at –80°C. Serial cryostat sections (6 μm) were prepared using tissue processor Leica CM3050. Lesion characterizations, including ORO staining of the thoracic-abdominal aorta and aortic root and staining for macrophages (anti-Mac2, BD Pharmingen, 553322, 1:900), T cells (anti-CD4, BD Pharmingen, 553043, 1:90; anti-CD8, Chemicon, CBL1318, 1:100), and VSMCs (SM-α-actin, MilliporeSigma, F-3777, 1:500), were performed as previously described (34, 54). The staining area was measured using Image-Pro Plus software, Media Cybernetics, and CD4^+^ and CD8^+^ cells were counted manually.

### Intimal RNA isolation from aorta tissue.

Isolation of intimal RNA from aorta was performed as previously described (34, 53). Briefly, aortas were carefully flushed with PBS, followed by intima peeling using TRIzol reagent (Invitrogen, 15596018). TRIzol was flushed for 10 seconds followed by a 10-second pause, then for another 10 seconds flushed, collected in an Eppendorf tube (~300–400 μL total), and snap frozen in liquid nitrogen. The intima-specific isolation was assessed in a previous study (15) by qPCR showing enrichment of endothelial marker CD31 in the intima fraction compared with media/adventitia fraction.

### Lipid profile analysis.

Lipid profile was measured as previously described (34). Briefly, triglyceride levels were determined using Infinity Triglycerides Liquid Stable Reagent (Thermo Fisher Scientific). Total cholesterol was measured using the Infinity Cholesterol Reagent (Thermo Fisher Scientific), and HDL-cholesterol was measured by colorimetric assay (BioAssay Systems, EnzyChrom HDL). LDL-cholesterol levels were calculated using the following formula: LDL = total cholesterol – HDL-cholesterol – (triglycerides/5). Standards were purchased from Pointe Scientific, Inc.

### Pig atherosclerotic samples.

The study protocol included 15 male hypercholesterolemic Yorkshire swine (Pine Acres Farm) that were placed on an HCD for up to 60 weeks. Detailed sectioning of 3 mm coronary artery segments was performed so that the gene sequencing samples were derived from the exact same portions of the coronary artery plaques used for the histology and IHC analyses. Histology and IHC analyses included H&E, van Gieson elastin staining, α–smooth muscle actin, ORO staining, picrosirius red staining, and CD31 and CD45 cells as described previously (15, 55).

### Human atherosclerotic specimens.

RNA was isolated from human carotid atherosclerotic lesions that were obtained from the Division of Cardiovascular Medicine, Brigham and Women’s Hospital in accordance with the IRB-approved protocol for use of discarded human tissues (protocol 2010-P-001930/2).

### Statistics.

For illustration of differentially expressed genes, GraphPad Prism software (V.7.0a) was used.

Data are shown as the mean ± SD. Statistical differences were calculated using unpaired 2-tailed Student’s *t* test or 1-way ANOVA with Bonferroni’s correction for multiple comparisons. A *P* value of less than 0.05 was considered significant.

### Study approval.

All protocols concerning animal use were approved by the IACUC at Brigham and Women’s Hospital and Harvard Medical School and conducted in accordance with the NIH’s *Guide for the Care and Use of Laboratory Animals* (National Academies Press, 2011). Studies were performed in *LDLR*^–/–^ male mice (The Jackson Laboratory, stock 002207) or in C57BL/6 mice (Charles River Laboratories, strain code 027). The IRB approved the use of discarded human tissues (protocol 2010-P-001930/2).

## Author contributions

MWF and VS conceived the hypothesis. VS, HZ, JBP, DY, and YT performed the experiments. VS, HZ, JBP, SH, GS, PS, PL, and MWF designed and interpreted the results. VS and MWF wrote the manuscript.

## Supplementary Material

supplemental data

## Version 1 10/06/2020

In-Press Preview

## Version 2 11/05/2020

Electronic publication
