# Supplementary material for: LncRNA VINAS regulates atherosclerosis by modulating NF-κB and MAPK signaling
Source: JCI Insight. 2020 Nov 5;5(21):e140627. doi: 10.1172/jci.insight.140627 (PMC7710319; doi:10.1172/jci.insight.140627)
Supplement: supplemental data [file jciinsight-5-140627-s073.pdf]

Supplementary Figures

LncRNA VINAS regulates atherosclerosis by modulating NF-κB and MAPK signaling

Viorel Simion[1#], Haoyang Zhou[1,2#], Jacob B. Pierce[1,3], Dafeng Yang[1,2], Stefan Haemmig[1], Yevgenia Tesmenitsky[1], Galina Sukhova[1], Peter H. Stone[1], Peter Libby[1], Mark W. Feinberg[1*]

Affiliations:

[1] Department of Medicine, Cardiovascular Division, Brigham and Women's Hospital, Harvard Medical School, Boston, MA, USA
[2] Department of Cardiology, The Third Xiangya Hospital of Central South University, Changsha, Hunan, China
[3] Feinberg School of Medicine, Northwestern University, Chicago, IL, USA

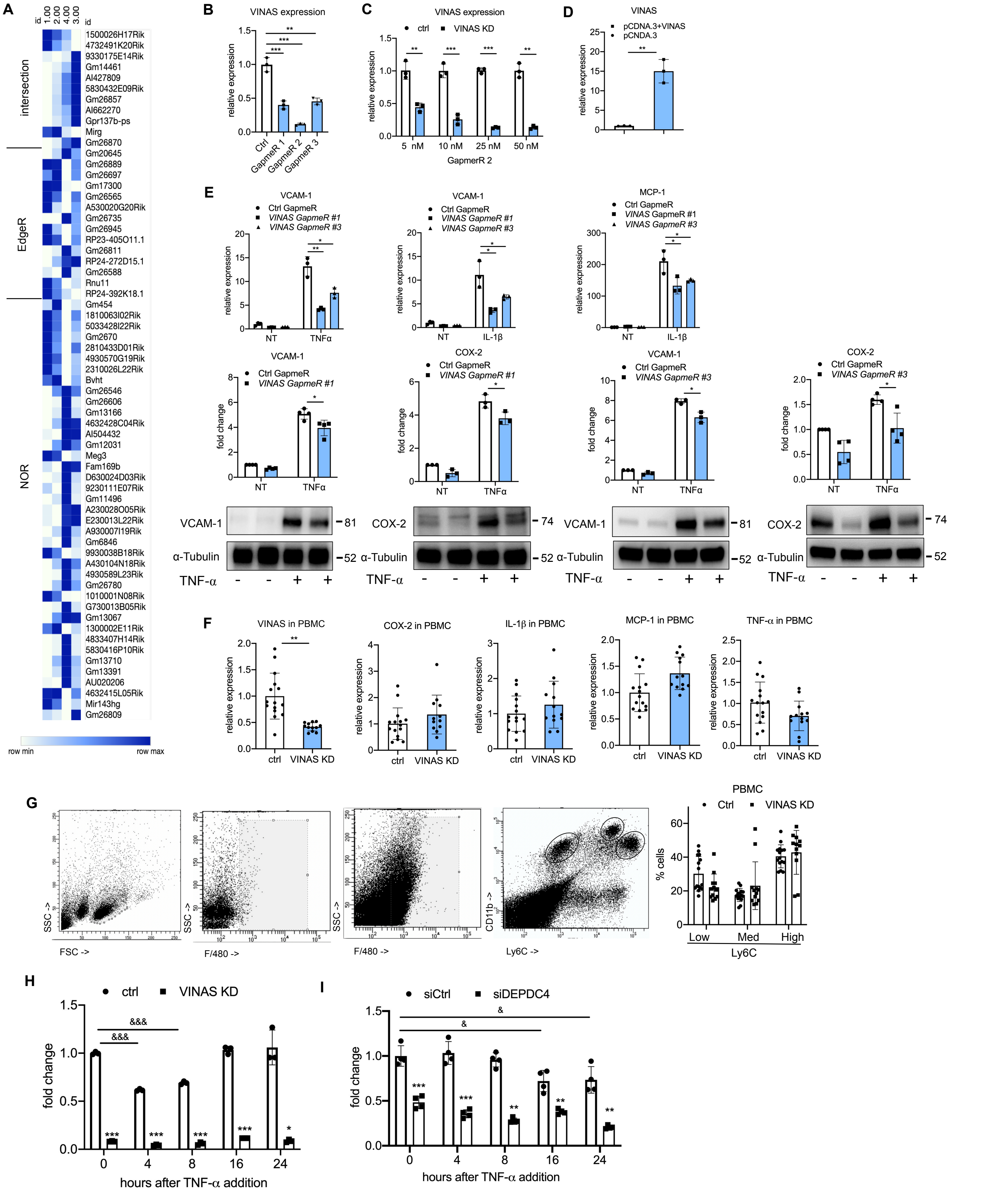

Supplementary Figure 1. Identification and characterization of lncRNA *VINAS*. **A.** LncRNA candidates identified by RNA-Seq with progression and regression of atherosclerosis in the aortic intima of LDLR-/- mice. **B.** *VINAS* silencing efficiency using three different *VINAS* gapmeRs in mouse ECs (n=3). **C.** *VINAS* silencing efficiency in mouse ECs using different concentrations of *VINAS* gapmeR #2 (n=3). **D.** *VINAS* overexpression using a pCDNA.3 plasmid in mouse ECs was quantified by RTqPCR (n=3). **E.** Anti-inflammatory effects of *VINAS* gapmeRs #1 and #3 on the gene expression of VCAM-1 (n=3) and MCP-1 (n=3) (upper panels) and on the protein expression of VCAM-1 (GapmeR #1, n=4; GapmeR #3, n=3) and COX-2 (GapmeR #1, n=3; GapmeR #3, n=4) (lower panels). **F.** *VINAS* silencing efficiency and expression of inflammatory markers COX-2, IL-1β, MCP-1 and TNF-α was assessed by RT-qPCR in the PBMCs from control gapmeR (n=6) and *VINAS* gapmeR groups of mice (n=5). **G.** Surface expression of Ly6C and CD11b in F4/80-sorted peripheral blood mononuclear cells (PBMCs) from LDLR-/- mice on HCD treated for 12 weeks with *VINAS* (n=13) or control gapmeRs (n=15). Mouse EC and HUVECs were first transfected with *VINAS* (**H**; n=3) and DEPDC4 (**I**; n=4) gapmeR and siRNA respectively; after 48 hours cells were treated with TNF-α (5 ng/ml) for different time points and RNA levels were quantified by RT-qPCR. For all panels, values are mean ± SD; *p < 0.05, **p<0.01; ***p<0.001 compared to GapmeR control; &<0.05, &&<0.01, &&&<0.001 compared to non-treated cells.

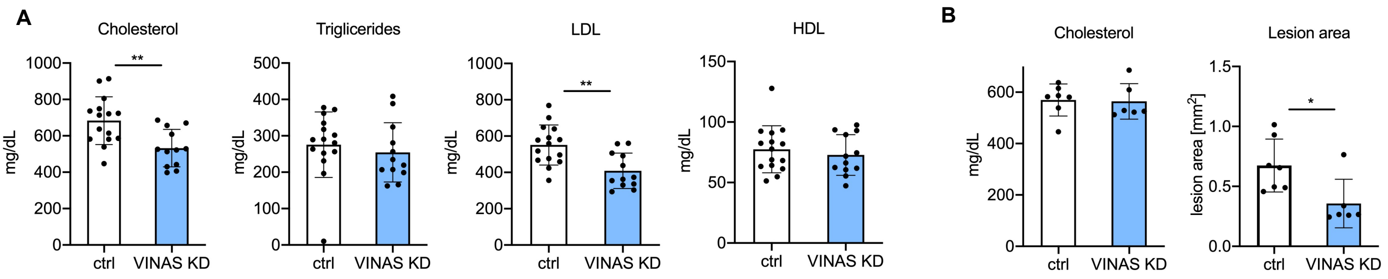

Supplementary Figure 2. Effects of *VINAS* knockdown on circulating lipids. **A.** Circulating lipid levels (total cholesterol, Triglycerides, LDL-C, and HDL) in the LDLR$^{-/-}$ HCD mice treated with control (n=15) or *VINAS* (n=13) gapmeRs after 12 weeks. **B.** When total cholesterol is normalized between LDLR$^{-/-}$ mice treated with control or *VINAS* gapmeRs, there is still a significant reduction of lesion area in the aortic sinus in the *VINAS* knockdown (KD) group. For all panels, values are mean ± SD; *p < 0.05, **p<0.01; ***p<0.001.

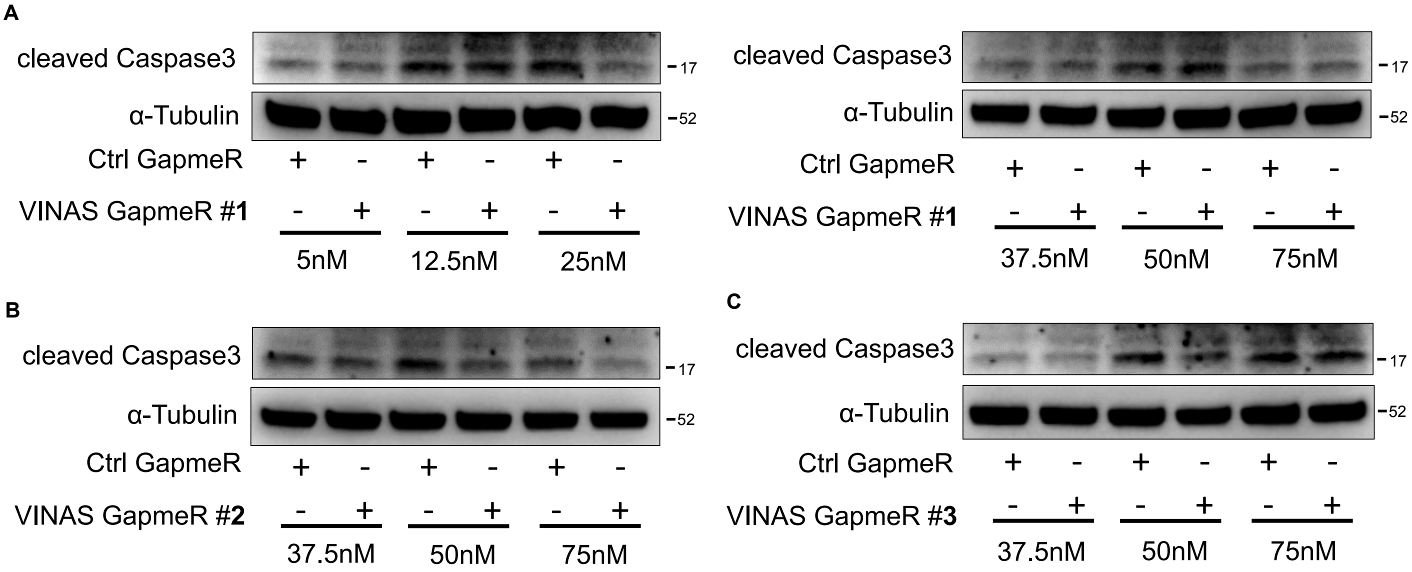

Supplementary Figure 3. Effect of *VINAS* knockdown on Caspase 3 cleavage. Mouse ECs were transfected with different concentrations of control and *VINAS* gapmeR #1, #2, #3, and Caspase 3 cleavage was assessed in the cell lysates by Western Blot.

A

M. musculus	TGGAAACAACAAACAGTCACATCT--CCTTAAATGGATCCACTTTCCGATCTTTGAAAATACTTTAGAGCCTCCAGTTAAAGTGTAAACTTCCCAGCTAAGTGAAGATGAAGATCTTGTCATTTCTAACACTTGTATAAAAAGAGAGGTTATTCAAA
H. sapiens	TGGAAAGAACAAACATTATTATGTCTTCTTCAATTGATTCACCTTCCATTCTTGGACAATATTTTGGAGCCTCCAGTTAAAACACAAAATCTTCAACTAAACAAAGAGGAAGATCTTGTTATCACTAACACTTGCCTAGACAGAGAACTTATTCCAA
P. troglodytes	TGGAAAGAACAAACATTATTATGTCTTCTTCAATTGATTCACCTTCCTTTCTTGGACAATATTTTGGAGCCTCCAGTTAAAACACAAAATCTTCAATTAAACAAAGAGGAAGATCTTGTTATTACTAACACTTGCCTAGACAGAGAACTTATTCCAA
M. mulatta	TGGAAAGAACAAACATTATTATGTCTTCTTTGATCGATTCACCTTCCATTCTTGGACAATATTTTGGAGCCTCCAGTTAAAACACAAAATCTTCTATTAAACAAAGAGGAAGATCTTGTTACCGCGAATACTTGCCTAGACAGAAAACTTATTCCAA
A. nancymaae	TGGAAAAAACAAACATTATTATGTATTCTTCAATTGATTCACCTTCCATTCTTGGAAAATGTTTTGAAGCCTCCAGTTAAAACACAAAATCTTCAATTAAACAAAGAGGAAAATCTTGTTATCACTAACACTTGCCTAGACAGAGAACTTACTCCAA
P. abelii	TGGAAAGAACAAACATTATTATGTCTTCTTCAATTGATTCACCTTCCATTCTTGGACAATATTTTGGAGCCTCCAGTTAAAACACAAAATTTTCAGTTAAACAAAGAGGAAGATCTTGTTATCACTAACACTTGCCTAGACAGAGAACTTATTCCAA
P. paniscus	TGGAAAGAACAAACATTATTATGTCTTCTTCAATTGATTCACCTTCCTTTCTTGGACAATATTTTGGAGCCTCCAGTTAAAACACAAAATCTTCAATTAAACAAAGAGGAAGATCTTGTTATTACTAACACTTGCCTAGACAGAGAACTTATTCCAA
Bos indicus	TGGAAAACAACAAACATGTTATGTATTCTTCAACTGATTCACCTTCCATTCTTGGAAAGTATTTCGGAACCCCTAGTTAAAACACAAATTCTTCAWTTAAATAGAGAGGAAGATCTTGTANTYGCAAACACTTAAATAGACAGAGAGGTGATTCCA
E. caballus	TGGAAACAACAAACATTGTTACATATTCTTCAATTGATTCACCTTCCATTCTTGGAAAATATTTTGGAGCCTCCAGTTAAAACACAAAATCATCAATTAAGTAAAGAGGAAGATCTCATTATCTCAAACACTTGTCTAGACAGAGAGATTATTCCAA
O. cuniculus	TGGAAACAACAAACATTGTTATGTATTCTCCAACTGATTCATCTTCCATTCTTGGAAAATATTTTAGAACCTCCAATTAAAGCACAAAATCTTCAATTAAGTAAGGAGGAAGATCTGGTTATCTCTAACACTTGCCTGGACAGAGAGCTTATTCCAA
C. mydas	TGGAAACAACAGACTCTGTTACGGCACTGCAGTTAATTGATCTTCCAGTCCTGGAAAATATTTTGGAGTCTCCAAACAAGAGGAAAAATGATCATCTTGGCAAAGAAGAAGATCTGATTATCTCAAATACTTTCCTTGACAGAGAGGTTACATGCA

B

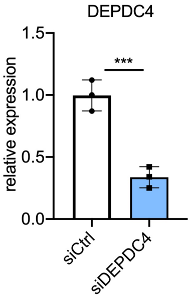

Supplementary Figure 4. DEPDC4 is evolutionary conserved across species. A. Sequence similarity across species at the *VINAS* / DEPDC4 gene locus. **B.** DEPDC4 silencing efficiency using specific siRNA (n=3). Values are mean ± SD; *p < 0.05, **p<0.01; ***p<0.001.

Table S1. Primers sequences

Primers	forward	reverse
VINAS	TAGGAAGCCCGAGTTTCTGGA	GTTTCCAGATGTCCTTCACAGC
DEPDC4	CCAGGAACCGTAGAGATGGC	CCACTTGGGCCTGAAGAGAG
GAPDH	AGGTCGGTGTGAACGGATTTG	TGTAGACCATGTAGTTGAGGTCA
COX-2	TGTGACTGTACCCGGACTGG	TGCACATTGTAAGTAGGTGGAC
MCP-1	GCTGGAGCATCCACGTGTT	ATCTTGCTGGTGAATGAGTAGCA
TNF-α	CTGGATGTCAATCAACAATGGGA	ACTAGGGTGTGAGTGTTTTCTGT
VCAM-1	CAACATGTGGCTCTGGGAAG	GCCAAACACTTGACCGTGAC
ICAM-1	TTCTCATGCCGCACAGAACT	TGTCGAGCTTTGGGATGGTA
E-selectin	ATGCCTCGCGCTTTCTCTC	GTAGTCCCGCTGACAGTATGC
MCP-1	TTAAAAACCTGGATCGGAACCAA	GCATTAGCTTCAGATTTACGGGT
COX-2	CATCCCCTTCCTGCGAAGTT	CATGGGAGTTGGGCAGTCAT
U6	CTCGCTTCGGCAGCACA	AACGCTTCACGAATTTGCGT
IL-1β	ATGCCACCTTTTGACAGTGATG	AGCTTCTCCACAGCCACAAT
COX2	TTCAACACACTCTATCACTGGC	AGAAGCGTTTGCGGTACTCAT

Table S2. Transcription factors binding to *VINAS* and DEPDC4 promoters

**VINAS**	**DEPDC4**
Elk-1	AP-2
Sp1	AP-2-alpha
Pegasus	AP-2-gamma
Sp1	AP-2
SIF	AP-2-alpha
E4F1	AP-2-gamma
AP-2-alpha	Ets

AP-2	SIF
AP-2-gamma	E4F1
AP-2-alpha	AP-2
AP-2	Pax4-PD
SIF	Sp1
PEA2	TEF
AP-2	AP-2-alpha
Pegasus	AP-2-gamma
AP-2-alpha	Ap-2
AP-2-gamma	STAT1-hs
AP-2	Pax4-PD
RAR	ZNF217
ARP-1	COUP-TF
H3abp	AP-2
AP-1	TFII-I
AP-2-alpha	AP-2-alpha
H4TF2	Pegasus
AP-2	AP-2-alpha
Pegasus	AP-2-gamma
AP-2-alpha	AP-2
AP-2-gamma	AP-2-alpha
AP-2-alpha	AP-2-gamma
AP-2-gamma	Nkx-3.2
AP-2	Elk-1
AP-2	COUP-TF
AP-2-alpha	Sp1

AP-2-gamma	Elk-1
AP-2	AP-2
AP-2-alpha	Sp1
Sp1	Sp1
Pegasus	Hox15
ENKTF1	AP-2-alpha
E4F1	AP-2
Pegasus	AP-2-alpha
STAT1-hs	Ets
STAT1-hs	Ets
Pegasus	Elk-1
STAT1-hs	ENKTF1
Ets	EGR-1
Ets	AP-2
H3abp	AP-2-alpha
ATF-CREB	AP-2-gamma
TEF	Pegasus
LRF-1	Pegasus
E4F1	Sp1
E4F1	Pax4-PD
LRF-1	ZNF219
Erg	H4TF2
STAT1-hs	AP-2

ATF-CREB	Pegasus
H3abp	STAT5A-hs
EF-1	STAT6-hs
EF-1A	MBF-1
E1A-F	Ets
Net_SAP2	AP-2-alpha
Elk-1	AP-2-gamma
STAT1-hs	AP-2
HNF-4	AP-2-alpha
Pegasus	AP-2-gamma
AP-2	H3abp
AP-2-alpha	ATF-CREB
AP-2-gamma	TEF
Pegasus	LRF-1
Sp1	E4F1
GCF	E4F1
AP-2	LRF-1
AP-2-alpha	Erg
AP-2-gamma	STAT1-hs
	ATF-CREB
	H3abp
	Ets
	EF-1A
	E1A-F
	PEA3
	Net_SAP2

	Elk-1
	Sp1
	STAT1-hs
	CP2
	AP-2-alpha
	AP-2-gamma
	AP-2
	E2A
	Sp1
	Pax4-PD
	ZNF219
